# Chronic liver disease not a significant comorbid condition for COVID-19

**DOI:** 10.1038/s41598-021-91238-8

**Published:** 2021-06-03

**Authors:** Jiahao Lin, Bingting Bao, Nigar Anjuman Khurram, Kasey Halsey, Ji Whae Choi, Lesan Wang, Thi My Linh Tran, Wei-Hua Liao, Michael D. Feldman, Paul J. Zhang, Jing Wu, Harrison X. Bai

**Affiliations:** 1 Department of Radiology, Second Xiangya Hospital, Central South University, Changsha, 410011 Hunan China; 2 Xiangya Medical School, Central South University, Changsha, 410013 Hunan China; 3 Department of Pathology, University of California, San Francisco, 91413 USA; 4 Department of Diagnostic Imaging, Rhode Island Hospital, Philadelphia, PA 19104 USA; 5 Warren Alpert Medical School, Brown University, Rhode Island Hospital, Providence, RI 02903 USA; 6 Department of Epidemiology and Health Statistics, Xiangya School of Public Health, Central South University, Changsha, 410008 Hunan China; 7 Department of Radiology, Xiangya Hospital, Central South University, Changsha, 410008 Hunan China; 8 Department of Pathology, University of Pennsylvania, Philadelphia, PA 19104 USA

**Keywords:** Hepatology, Liver diseases

## Abstract

To explore the role of chronic liver disease (CLD) in COVID-19. A total of 1439 consecutively hospitalized patients with COVID-19 from one large medical center in the United States from March 16, 2020 to April 23, 2020 were retrospectively identified. Clinical characteristics and outcomes were compared between patients with and without CLD. Postmortem examination of liver in 8 critically ill COVID-19 patients was performed. There was no significant difference in the incidence of CLD between critical and non-critical groups (4.1% vs 2.9%, *p* = 0.259), or COVID-19 related liver injury between patients with and without CLD (65.7% vs 49.7%, *p* = 0.065). Postmortem examination of liver demonstrated mild liver injury associated central vein outflow obstruction and minimal to moderate portal lymphocytic infiltrate without evidence of CLD. Patients with CLD were not associated with a higher risk of liver injury or critical/fatal outcomes. CLD was not a significant comorbid condition for COVID-19.

## Introduction

The Coronavirus disease-19 (COVID-19), caused by the severe acute respiratory syndrome CoV-2 coronavirus (SARS-CoV-2), has posed a critical threat to global health. As of November 8, 2020, over 49.7 million COVID-19 patients with over 1.2 million deaths globally have been confirmed^1^.

Mortality rates of this pandemic disease were reported to be 2–6%^2^, which can be associated with comorbidities such as hypertension, diabetes and cardiovascular disease^3^. In addition, previous studies have shown a close association between liver injury and COVID-19 severity^4^. However, the impact of chronic liver disease (CLD) as a comorbidity on the outcome of COVID-19 remains controversial^5–8^. Patients with CLD were assumed to be susceptible for developing COVID-19 and liver injury due to their immunocompromised status. However, the overall prevalence of CLD in COVID-19 was reported to be 3% by a recent meta-analysis^9^, which was low compared to other comorbidities^3^. Worse outcomes were either reported to be associated with CLD or not seen in patients with CLD^5–8^.

Liver injury in COVID-19 or the disease itself can be related to an immune reaction. The host immunity of patients with CLD is likely compromised^10,11^. However, this may serve a protective function rather than being harmful, and may explain controversial findings in the existing literature. With the goal of exploring the complex role of CLD in COVID-19, this study investigated factors associated with critical outcomes for COVID-19 and compared the clinical characteristics and outcomes between patients with and without CLD.

## Methods

### Study design and participants

A retrospective observational cohort study included patients diagnosed with COVID-19 from one large academic center in the United States from March 16, 2020 to April 23, 2020. Detailed data sources are shown in Supplementary Fig. S1. A total of 1439 consecutively hospitalized patients were included in the analysis. The study was approved and the requirement for informed consent was waived by Institutional Review Boards of Hospital of University of Pennsylvania. All methods were carried out in accordance with relevant guidelines and regulations.

### Data collection

Clinical and laboratory data were collected from the electronic medical records. For each patient, the extracted data were divided into four categories including demographics (age and sex), comorbidities (hypertension, diabetes, cardiac or cerebrovascular disease, malignancy, chronic liver or kidney disease), liver function tests, and clinical outcomes. CLD was defined as progressive deterioration of liver functions for more than 6 months before admission^12^. Liver function test included alanine aminotransferase (ALT, normal range: 6–45 U/L), aspartate aminotransferase (AST, 10–42 U/L), alkaline phosphatase (ALP, 34–104 U/L), total protein (TP, 6.0–8.0 G/DL), albumin (3.5–5.0 G/DL), total bilirubin (TB, 0.2–1.3 MG/DL), and direct bilirubin (DB, 0–0.3 MG/DL). All liver function tests available before and after the diagnosis of COVID-19 were recorded to assess dynamic change. Liver injury was defined as any parameter above the normal upper limit, based on the worst liver function test result recorded during hospitalization. The following outcome measures were determined: hospital mortality, use of mechanical ventilation, and intensive care unit (ICU) admission. Patients were then classified into critical and non-critical groups. Patients were defined as critical if they had any of the following outcomes: death, use of mechanical ventilation, or admission to the ICU. Otherwise, they were defined as non-critical. Lastly, postmortem histologic examination of liver was performed in eight COVID-19 patients who died consecutively during the months of May and early June 2020 in a tertiary care center in the United States.

### Statistical analysis

Outcome and baseline characteristics were compared between critical and non-critical groups using chi-square test or Student’s t test. Kaplan–Meier survival analysis with log-rank test was performed to compare overall survival and time to mechanical ventilation/ICU admission in COVID-19 patients with or without CLD. Multivariable Cox regression analysis for the association between base characteristics and adverse outcome was performed. All analyses were performed using SPSS 22.0 (SPSS Inc., Chicago, IL).

## Results

Among 1439 patients included, 415 (28.8%) patients had critical outcomes. Patients in critical group were older than those in non-critical group (mean age, 66.8 years vs 50.5 years, *p* < 0.001 ), with higher incidence of comorbidities including hypertension (69.4% vs 45.5%, *p* < 0.001), diabetes (47.0% vs 26.2%, *p* < 0.001), cardiovascular disease (42.7% vs 18.7%, *p* < 0.001), and malignancy (14.9% vs 6.7%, *p* < 0.001) (Table 1). Multivariable Cox regression analysis showed that patients older than 50 years had a significantly higher risk of critical outcomes (HR, 0.649; 95% CI, 0.451–0.933; *p* = 0.020). Comorbidities including hypertension (HR, 0.683; 95% CI, 0.485–0.963; *p* = 0.030), cardiovascular disease (HR, 0.698; 95% CI, 0.537–0.907; *p* = 0.007), and malignancy (HR, 0.613; 95% CI, 0.446–0.841; *p* = 0.002) were independent predictors of critical outcomes in multivariable Cox regression analysis (Table 2).

**Table 1:** Clinical characteristics of COVID-19 patients stratified into critical or non-critical groups.

Clinical and laboratory	Total (n = 1439)	Non-critical (n = 1024)	Critical^†^ (n = 415)	*p* Value
**Age, mean ± SD, years**	55.2 ± 19.9	50.5 ± 19.4	66.8 ± 15.7	< 0.001
**Sex**				
Male	723 (50.2)	495 (48.3)	228 (54.9)	0.023
Female	716 (49.8)	529 (51.7)	187 (45.1)	
**Comorbidities**				
Cardiovascular disease	368 (25.6)	191 (18.7)	177 (42.7)	< 0.001
Chronic kidney disease	198 (13.8)	114 (11.1)	84 (20.2)	< 0.001
Chronic liver disease	47 (3.3)	30 (2.9)	17 (4.1)	0.259
Diabetes	463 (32.2)	268 (26.2)	195 (47.0)	< 0.001
Hypertension	754 (52.4)	466 (45.5)	288 (69.4)	< 0.001
Malignancy	131 (9.1)	69 (6.7)	62 (14.9)	< 0.001
**Abnormal liver function tests‡**				
ALT	289 (64.8)	128 (62.8)	161 (66.5)	0.494
AST	420 (94.1)	186 (91.2)	234 (96.6)	0.040
AKP	205 (46.0)	84 (41.2)	121 (50.0)	0.114
TP	247 (55.8)	69 (34.0)	178 (74.1)	< 0.001
Albumin	357 (80.9)	128 (63.3)	229 (96.0)	< 0.001
TB	57 (12.7)	15 (7.5)	42 (17.0)	0.010
DB	105 (29.0)	33 (20.7)	72 (35.6)	0.008
**Liver injury§**	446 (50.5)	204 (38.3)	242 (69.0)	< 0.001

SD, standard deviation; ALT, alanine aminotransferase; AST, aspartate aminotransferase; ALP, alkaline phosphatase; TP, total protein; TB, total bilirubin; DB, direct bilirubin.

^†^Patients were classified into critical and non-critical groups. Patients were defined as critical if they had any of the following outcomes: death, use of mechanical ventilation, or admission to the ICU. If they did not, they were defined as non-critical.

^‡^The number and percentage for all the lab values are for patients with abnormal lab values (i.e., above the normal upper limit).

^§^Liver injury was defined as any parameter above the normal upper limit (including ALT, 6–45 U/L; AST, 10–42 U/L; ALP, 34–104 U/L; TP, 6.0–8.0 G/DL; albumin, 3.5–5.0 G/DL; TB, 0.2–1.3 MG/DL; DB, 0–0.3 MG/DL), based on the worst liver function test result recorded during hospitalization.

**Table 2:** Multivariable Cox regression for clinical outcomes in COVID-19 patients.

Variable	β	HR (95% CI)	*p*
Sex	− 0.186	1.204 (0.936–1.548)	0.148
Age (≤ 50/ > 50)	0.432	0.649 (0.451–0.933)	0.020
Chronic kidney disease	0.109	0.897 (0.671–1.200)	0.464
Chronic liver disease	− 0.161	1.175 (0.702–1.965)	0.540
Hypertension	0.381	0.683 (0.485–0.963)	0.030
Diabetes	0.240	0.786 (0.604–1.023)	0.074
Cardiovascular disease	0.359	0.698 (0.537–0.907)	0.007
Malignancy	0.490	0.613 (0.446–0.841)	0.002
Liver injury	0.919	2.506 (1.913–3.285)	< 0.001

Among 1439 COVID-19 positive patients, 47 (3.3%) patients with CLD were identified, with 36 chronic hepatitis B/C, 7 non-alcoholic steatohepatitis (NASH), 5 alcoholic cirrhosis, 4 combination of chronic hepatitis B/C and NASH or alcoholic cirrhosis or hepatocellular carcinoma, and 1 other specified disorders of liver. The detailed clinical information of these 47 patients with CLD are shown in Supplementary Table S1. There was no significant difference in the incidence of CLD between critical and non-critical groups (4.1% vs 2.9%, *p* = 0.259). Among 47 patients with CLD, 35 patients had a record of liver function tests, and 17(63.0%) patients had critical outcomes. There was no significant difference between clinical variables and adverse outcomes (Supplementary Table S2). Among 1439 patients included, 901 patients had a record of liver function tests, 446 of whom developed acute liver injury as judged by new abnormal liver function test during hospitalization. The incidence of acute liver injury was higher in the critical group than non-critical group (69.0% vs. 38.3%, *p* < 0.001). Liver injury was an independent predictor of critical outcomes (HR, 2.506; 95% CI, 1.913–3.285; *p* < 0.001) in multivariable Cox regression analysis.

**Figure 1:**
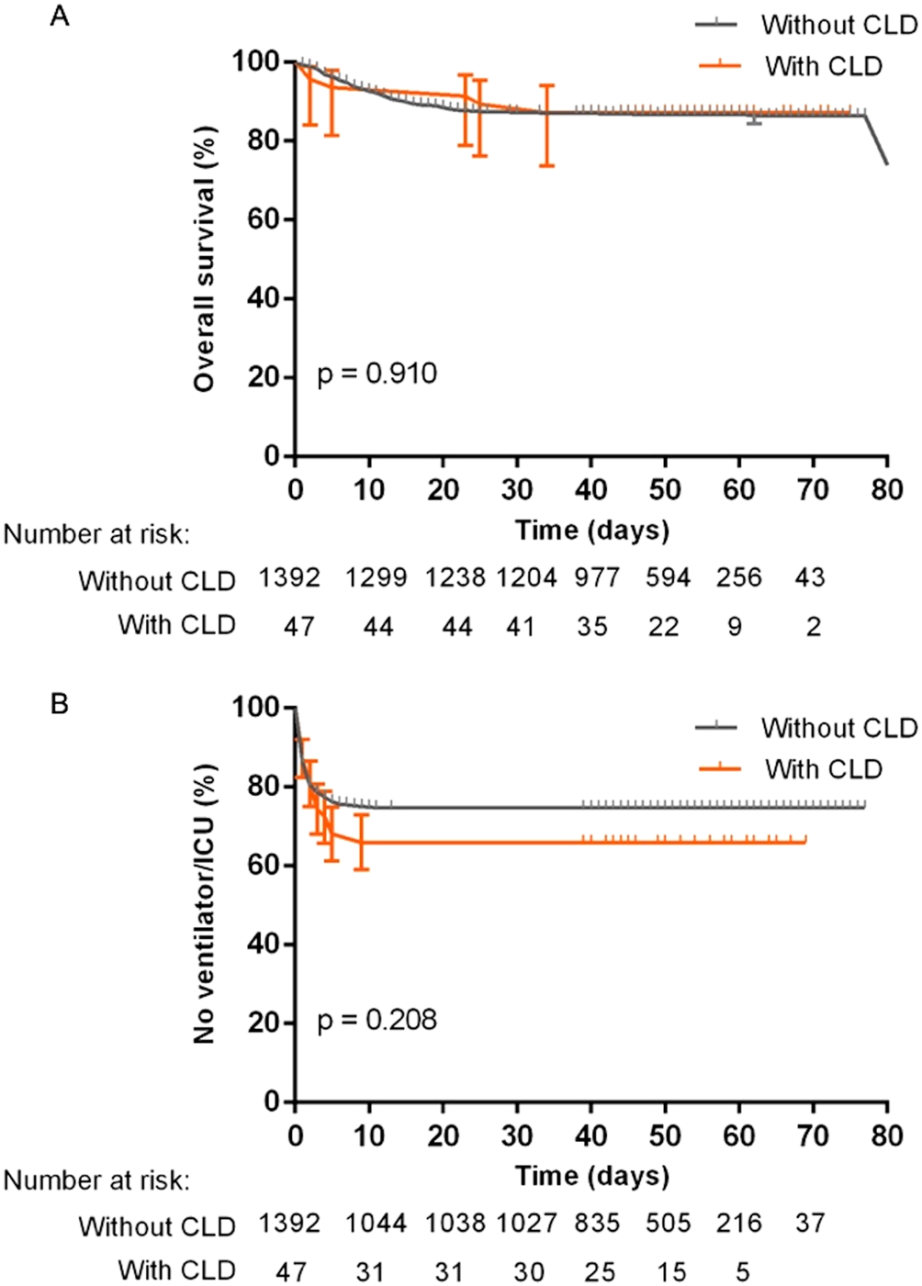
Kaplan–Meier survival curves of (**A**) overall survival, and (**B**) time to mechanical ventilation/ICU admission in COVID-19 patients with CLD or without CLD.

There was no significant difference in the incidence of acute liver injury (65.7% vs 49.7%, *p* = 0.065) or mean time to development of acute liver injury (3.30 days vs 3.26 days, *p* = 0.968) between CLD and non-CLD groups (Supplementary Table S3). There was also no significant difference between the CLD and non-CLD groups in overall survival (log-rank *p* = 0.910) or time to mechanical ventilation/ICU admission (log-rank *p* = 0.208) (Fig. 1).

**Figure 2:**
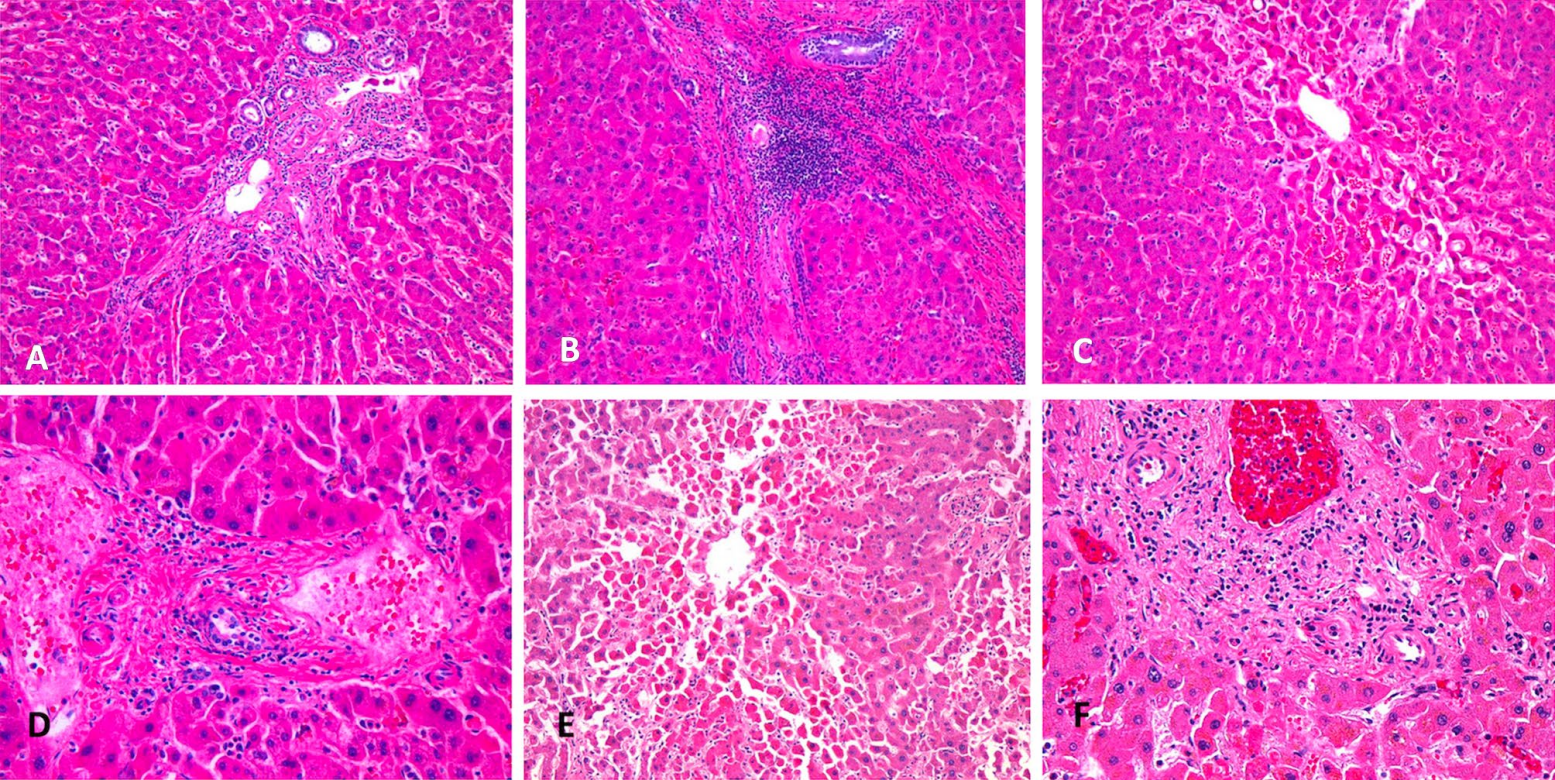
Postmortem liver findings in COVID-19. (**A**), (**B**) and (**C**) from patient 5, show minimal (**A**) to moderate (**B**) portal lymphocytic infiltrate and central vein outflow obstruction injury (**C**); (**D**) from patient 3, shows minimal portal lymphocytic infiltrate and fibrin thrombi; (**E**) and (**F**) from patient 7, show extensive central vein outflow obstruction associated with centrilobular necrosis (**E**) and mild portal lymphocytic infiltrate (**F**).

In postmortem liver histologic evaluation, the most common finding was variable degree of lymphocytic portal triaditis, followed by variable degree of central vein out flow obstruction and injury and focal fibrin thrombi. No bile duct injury was detected. Concordant with history, there was no evidence of CLD such as cirrhosis, chronic hepatitis, and steatosis in all eight patients (Fig. 2; Supplementary Table S4).

## Discussion

With data from one large academic center in the United States, the current study demonstrated that the incidence of CLD in COVID-19 (3.3%) was low and within the range of previous studies^4,5,9,13,14^. CLD was a very uncommon comorbidity for COVID-19 patients compared to other comorbidities and it also did not appear to be associated with critical outcomes. The incidence of CLD (3.3%) was even lower than the prevalence of hepatitis B infection in the US adult population (4.3%)^15^. The incidence of alcoholic hepatitis was also lower in our patients (< 1%) than in the general US population (8%)^16^. These findings concurred with the observation that no evidence of CLD was found in eight postmortem liver autopsies, while liver injury was common in critical patients due to variable severity of central vein out flow obstruction and mild portal lymphocytic triaditis. Central vein outflow associated pathology is likely due to right heart failure commonly seen in these fatal cases while the significance and biology of lymphocytic triaditis is unknown.

Similar to other studies, older age, and comorbidities including hypertension, acute liver injury, malignancy and cardiovascular diseases were associated with critical outcomes. The incidence of liver injury was higher in critically ill patients than in non-critically ill patients. However, none of the above indicators was associated with critical outcomes in sub analysis of patients with CLD, and liver injury was not associated with a history of CLD in hospitalized COVID-19 patients. The time distribution of liver injury also suggested that acute liver injury generally occurred earlier and was not affected by the side effects of treatment. The main results in the present study are consistent with a previous study, which demonstrated that outcomes were not worse in 47 patients with CLD and COVID-19 than those without CLD and COVID-19 using data from seven Chinese studies^8^. Another study from China by Guan et al. showed that only one out of 23 COVID-19 patients (2.1%) with Hepatitis B infection had a severe outcome^5^. Fan et al. also showed that there was no statistical difference in the proportion of chronic hepatitis B/C between an abnormal liver function group and a normal liver function group^17^. Interestingly, a limited number of studies and a recent meta-analysis reported findings contradictory to those herein. Singh et al. compared the outcomes of patients with and without a preexisting liver disease by using the TriNetX (Cambridge, MA) Research Network, and found that patients with a preexisting liver disease were at increased risk for mortality compared to patients without liver disease^7^. Ji et al. demonstrated that patients with NAFLD had a higher risk of liver injury and disease progression^6^. A meta-analysis conducted by Kovalic et al. used data from 73 pooled clinical studies to conclude that CLD is associated with more severe COVID-19 infection and higher mortality in COVID-19 patients^18^.

The most likely explanation for the high prevalence of positive associations found in the literature between CLD and COVID-19 severity is that patients with CLD in the meta-analysis probably have other comorbidities that contributed to the higher severity and mortality. This was not investigated in the meta-analysis because multivariate analyses were not performed. Publication bias could also be in favor of these positive findings. Another possible reason for contradictory results in the literature could be the different composition of CLD types in COVID-19 patients. Kovalic et al. mentioned that most of the studies included in their meta-analysis did not categorize their CLD patients based on specific disease etiologies of CLD nor did they come with a set definition for CLD^18^, which makes it more difficult to pinpoint if the positive association they found between CLD and COVID-19 severity is driven by one type of CLD or various types or CLD at all. It is known that the majority (77%, 36/47) of our patients from the United States had chronic hepatitis B/C, while patients with chronic hepatitis B/C in the study by Singh et al. only accounted for 21% of the COVID-19 cohort^7^—the worse outcomes observed in CLD patients with COVID-19 from their study could have been due to etiologies of CLD other than hepatitis B/C. We speculate that the comorbid mechanism of liver injury by various hepatitis viruses in COVID-19 patients is different from that of other types of CLD. Another possible explanation is altered immune status of unknown etiology in some patients with CLD. This is supported by the observation of lymphocytic triaditis during postmortem evaluation of critical COVID-19 patients. Although the liver injury in these 8 patients did not seem to contribute significantly to their demise, the potential long-term effect of this possible viral related liver injury in COVID-19 patients remains unknown. These observations may support a possible protective role of some CLD diseases in COVID-19 through alternating the host innate immunity against the virus^19^.

Several limitations in the present study are worth noting. First, this is a retrospective study from a single academic center in the United States. Second, all patients enrolled in the study were inpatient and possible selection bias cannot be eliminated because outpatients can also suffer from CLD. Since the incidence of CLD in outpatients tends to be similar if not lower than that in inpatients and outpatients tend to less ill^20^, it is likely that adding outpatients may further support our findings. To better define the impact of CLD on COVID-19, future studies will focus on subgroup analysis of CLD with different etiologies and severities in a larger, prospective cohort.

In conclusion, patients with CLD were not associated with higher risk of liver injury or critical outcomes. In hospitalized COVID-19 patients, CLD did not appear to be a premorbid or comorbid condition to COVID-19. These findings can inform healthcare providers’ determination of risk factors for critical outcomes and their subsequent allocation of healthcare resources during the current COVID-19 pandemic. Further studies with a large cohort size and subgroup analysis are needed to validate these findings.

## Supplementary Information

Supplementary Information.

## Data Availability

The datasets analyzed during the current study are available from the corresponding author on reasonable request.

## Abbreviations

CLD: Chronic liver disease
COVID-19: Coronavirus disease-19
SARS-CoV-2: The severe acute respiratory syndrome CoV-2 coronavirus
ICU: Intensive care unit
SD: Standard deviation
ALT: Alanine aminotransferase
AST: Aspartate aminotransferase
ALP: Alkaline phosphatase
TP: Total protein
TB: Total bilirubin
DB: Direct bilirubin
NASH: Non-alcoholic steatohepatitis
NAFLD: Nonalcoholic fatty liver disease
