# Supplementary material for: Chronic liver disease not a significant comorbid condition for COVID-19
Source: Sci Rep. 2021 Jun 3;11:11734. doi: 10.1038/s41598-021-91238-8 (PMC8175434; doi:10.1038/s41598-021-91238-8)
Supplement: Supplementary file 1 — Supplementary Information. [file 41598_2021_91238_MOESM1_ESM.pdf]

Chronic liver disease not a significant comorbid condition for COVID-19

Jiahao Lin[1+], M.D.; Bingting Bao[4+], M.S.; Nigar Anjuman Khurram[5], M.D.; Kasey Halsey[2,3], B.A.; Ji Whae Choi[2,3], B.A.; Lesan Wang[8], MD. PhD.; Thi My Linh Tran[2,3], B.S.; Wei-Hua Liao[6], M.D.; Michael D. Feldman[7], MD. PhD.; Paul J. Zhang[7], M.D.; Jing Wu[1*], M.D; Harrison X. Bai[2,3*], M.D..

1. Department of Radiology, Second Xiangya Hospital, Central South University, Changsha, Hunan 410011, China
2. Department of Diagnostic Imaging, Rhode Island Hospital, Philadelphia, PA 19104, USA
3. Warren Alpert Medical School, Brown University, Rhode Island Hospital, Providence, RI 02903, USA
4. Xiangya Medical School, Central South University, Changsha, Hunan 410013, China
5. Department of Pathology, University of California, San Francisco 91413, USA
6. Department of Radiology, Xiangya Hospital, Central South University, Changsha, Hunan 410008, China
7. Department of Pathology, University of Pennsylvania, Philadelphia, PA 19104, USA
8. Department of Epidemiology and Health Statistics, Xiangya School of Public Health, Central South University, Changsha, Hunan 410008, China

*corresponding author: wujingkiki622@gmail.com, Harrison X. Bai: harrison_bai@brown.edu

+These authors contributed equally to this study.

Supplementary

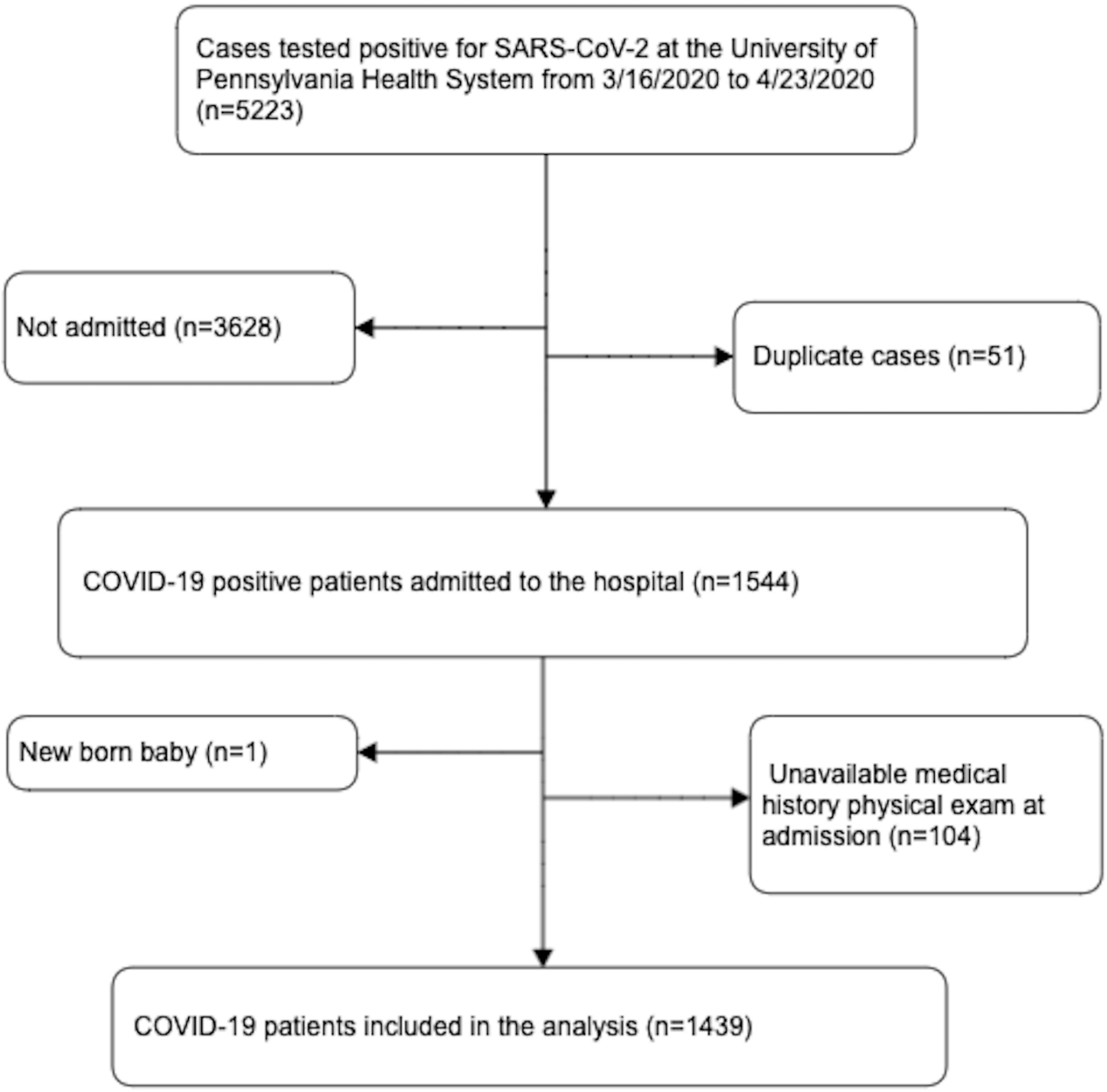

Supplementary Fig. S1. Flow chart of patient inclusion and exclusion

Supplementary Table S1. Clinical information of 47 COVID-19 patients with chronic liver disease

Patients	Age (years)	Gender	Diagnosis	Time (months)	HCVAb/ HBcAb	AKP before COVID(U/L)	Albumin before COVID(g/dL)	AST/ALT before COVID(U/L)	Histopathology	Image modality	Image finding			Disease stage
											Cirrhosis	Steatosis	Hepatomegaly	
1	81	Male	Chronic hepatitis C	22	reactive	59	3.5	38/37	na	na	na	na	na	1-Hepatitis
2	79	Female	Chronic hepatitis C	18	reactive	60	3	16/18	na	CT	no	no	no	1-Hepatitis
3	66	Male	Chronic hepatitis B	16	reactive	44	4.3	33/14	na	na	na	na	na	1-Hepatitis
4	60	Female	Chronic hepatitis C	13	reactive	37	2.2	45/29	na	US	no	yes	yes	1-Hepatitis
5	48	Male	Non-alcoholic steatohepatitis, Chronic hepatitis C	10	reactive	83	4.6	18/33	na	US	no	yes	no	1-Steatosis
6	79	Male	Chronic hepatitis C, Fibrosis of liver	82	reactive	112	4	15/11	Chronic hepatitis, consistent with hepatitis C virus infection, Grade 2, Stage 2	US	yes	no	no	3-Cirrhosis
7	35	Male	Chronic hepatitis C	8	reactive	115	4.1	57/111	na	na	na	na	na	1-Hepatitis
8	27	Male	Chronic hepatitis B	81	reactive	61	4.3	18/15	na	na	na	na	na	1-Hepatitis

9	32	Male	Chronic hepatitis B	7	reactive	50	4.3	66/58	na	na	na	na	na	1-Hepatitis
10	61	Male	Chronic hepatitis C, Hepatocellular carcinoma	62	reactive	88	4.5	17/13	na	MR	yes	no	no	4-Hepatocellular carcinoma
11	67	Male	Chronic hepatitis C	44	reactive	128	3.7	14/10	na	na	na	na	na	1-Hepatitis
12	59	Female	Chronic hepatitis C	70	reactive	145	4	17/18	na	na	na	na	na	1-Hepatitis
13	70	Male	Chronic hepatitis B, cirrhosis	78	reactive	49	4	30/30	na	US	yes	yes	no	3-Cirrhosis
14	25	Female	Chronic hepatitis C, cirrhosis	31	reactive	83	2.4	23/10	na	US	yes	yes	yes	3-Cirrhosis
15	65	Male	Fatty liver	39	na	74	4.1	25/28	na	US	no	yes	yes	1-Steatosis
16	84	Female	Fatty liver	104	na	55	3	14/14	na	na	na	na	na	1-Steatosis
17	62	Female	Alcoholic cirrhosis	86	na	204	2.9	68/20	na	CT	no	yes	no	3-Cirrhosis
18	69	Male	Chronic hepatitis C, cirrhosis	37	reactive	60	4.7	26/25	na	na	na	na	na	3-Cirrhosis
19	58	Male	Chronic hepatitis C, cirrhosis	43	reactive	53	3.7	19/17	na	US	yes	no	yes	3-Cirrhosis
20	62	Male	Chronic hepatitis B	8	reactive	46	3.5	65/37	na	na	na	na	na	1-Hepatitis

21	77	Female	Chronic hepatitis C, cirrhosis	15	reactive	69	2.6	9/5	na	na	na	na	na	3-Cirrhosis
22	62	Male	Chronic hepatitis C	94	reactive	79	3.3	15/9	na	na	na	na	na	1-Hepatitis
23	69	Female	Chronic hepatitis C	32	reactive	74	3.6	17/13	na	na	na	na	na	1-Hepatitis
24	72	Male	Chronic hepatitis C, cirrhosis, hepatocellular carcinoma	68	reactive	65	4.4	24/18	na	MR	yes	yes	yes	4-Hepatocellular carcinoma
25	62	Male	Chronic hepatitis C,cirrhosis	91	reactive	66	3.8	12/9	na	US	yes	yes	no	3-Cirrhosis
26	59	Male	Chronic hepatitis C	156	reactive	49	3.9	18/14	na	na	na	na	na	1-Hepatitis
27	42	Male	Chronic hepatitis B, cirrhosis	17	reactive	71	4.3	23/11	na	US	yes	no	no	3-Cirrhosis
28	74	Male	Chronic hepatitis C	48	reactive	62	3.3	18/15	na	na	na	na	na	1-Hepatitis
29	68	Female	Alcoholic cirrhosis	22	na	53	3.9	15/13	na	CT	no	no	no	3-Cirrhosis
30	72	Female	Chronic hepatitis B	88	reactive	79	2.6	24/17	na	na	na	na	na	1-Hepatitis
31	43	Male	Other specified disorders of liver	149	na	68	4.7	16/17	na	na	na	na	na	1-Hepatitis

32	60	Female	Hepatic steatosis	132	na	59	4.2	38/27	na	US	no	yes	yes	1-Hepatosteatosis
33	35	Female	Chronic hepatitis B	113	reactive	53	3.1	16/13	na	na	na	na	na	1-Hepatitis
34	54	Male	Hepatic steatosis	10	na	47	4.5	68/58	na	CT	no	yes	no	1-Hepatosteatosis
35	75	Female	Chronic hepatitis C	55	reactive	64	3.2	9/3	na	na	na	na	na	1-Hepatitis
36	52	Female	Alcoholic cirrhosis	89	na	78	3.9	14/7	na	na	na	na	na	3-Cirrhosis
37	41	Male	Chronic hepatitis B	49	reactive	39	4.4	16/21	na	US	no	yes	no	1-Hepatitis
38	75	Female	Hepatic steatosis	100	na	417	3.4	84/93	na	US	no	no	yes	1-Hepatosteatosis
39	36	Female	Hepatic steatosis	7	na	108	3.1	16/23	na	CT	no	yes	no	1-Hepatosteatosis
40	66	Male	Chronic hepatitis C	80	reactive	116	1.3	18/11	na	na	na	na	na	1-Hepatitis
41	58	Male	Chronic hepatitis C	124	reactive	na	na	na	na	na	na	na	na	1-Hepatitis
42	57	Male	Chronic hepatitis C	86	reactive	63	4.2	52/47	na	na	na	na	na	1-Hepatitis
43	68	Female	Alcoholic cirrhosis	22	na	53	3.9	15/13	na	CT	no	no	no	3-Cirrhosis
44	55	Male	Alcoholic cirrhosis	40	na	111	4.2	57/61	na	US	yes	no	no	3-Cirrhosis
45	58	Male	Chronic hepatitis C	191	reactive	38	2.7	22/14	Chronic hepatitis, grade 1 of 4, stage 1 of 4.	US	yes	no	no	3-Cirrhosis
46	70	Male	Chronic hepatitis C	189	reactive	50	4.4	13/11	na	US	yes	no	no	3-Cirrhosis

47	67	Female	Chronic hepatitis B, cirrhosis	17	reactive	301	2.9	41/27	na	CT	no	no	no	3-Cirrhosis

Abbreviations: AKP, alkaline phosphatase; ALT, alanine aminotransferase; AST, aspartate aminotransferase.

Supplementary Table S2. Clinical characteristics of COVID-19 patients with CLD stratified into critical or non-critical groups

Clinical and laboratory	Total (n=47)	Non-critical (n=30)	Critical† (n=17)	P value
Age, mean±SD, years	55.2±19.9	57.9±13.8	62.0±15.3	0.356
Sex				
Male	31 (66.0)	18 (60.0)	13 (76.5)	0.252
Female	16 (34.0)	12 (40.0)	4 (23.5)	
Comorbidities				
Cardiovascular disease	17 (36.2)	10(33.3)	7(41.2)	0.591
Chronic kidney disease	16 (34.0)	10(33.3)	6(35.3)	0.892
Diabetes	21 (44.7)	12(40.0)	9(52.9)	0.391
Hypertension	34 (72.3)	20(66.7)	14(82.4)	0.415
Malignancy	8 (17.0)	3(10.0)	5(29.4)	0.194
Abnormal liver function tests‡				
ALT	12(52.2)	3(30.0)	9(69.2)	0.100
AST	22(95.7)	9(90.0)	13(100.0)	0.435
AKP	12(52.2)	5(50.0)	7(53.8)	1.000
TP	11(47.8)	4(40.0)	7(53.8)	0.680
Albumin	15(65.2)	5(50.0)	10(76.9)	0.221
TB	4(17.4)	0(0.0)	4(30.8)	0.104
DB	5(21.7)	1(10.0)	4(30.8)	0.613
Liver injury§	23 (65.7)	10 (52.6)	13 (81.3)	0.152

Abbreviations: CLD, chronic liver disease; SD, standard deviation; ALT, alanine aminotransferase; AST, aspartate aminotransferase; ALP, alkaline phosphatase; TP, total protein; TB, total bilirubin; DB, direct bilirubin.

†: Patients were classified into critical and non-critical groups. Patients were defined as critical if they had any of the following outcomes: death, use of mechanical ventilation, or admission to the ICU. If they did not, they were defined as non-critical.

‡: The number and percentage for all the lab values are for patients with abnormal lab values (i.e., above the normal upper limit).

§: Liver injury was defined as any parameter above the normal upper limit (including ALT, 6-45 U/L; AST, 10- 42 U/L; ALP, 34-104 U/L; TP, 6.0-8.0 G/DL; albumin, 3.5-5.0 G/DL; TB, 0.2-1.3 MG/DL; DB, 0-0.3 MG/DL), based on the worst liver function test result recorded during hospitalization.

Supplementary Table S3. Clinical characteristics of COVID-19 patients with or without CLD

Clinical and laboratory	Total (n=1439)	No CLD(n=1392)	With CLD(n=47)	P value
Age, mean±SD, years	55.2±19.9	55.1±20.0	59.4±14.3	＜0.001
Sex				
Male	723 (50.2)	692 (49.7)	31 (66.0)	0.028
Female	716 (49.8)	700 (50.3)	16 (34.0)	
Comorbidities				
Cardiovascular disease	368 (25.6)	351 (25.2)	17 (36.2)	0.090
Chronic kidney disease	198 (13.8)	182 (13.1)	16 (34.0)	＜0.001
Diabetes	463 (32.2)	442 (31.8)	21 (44.7)	0.062
Hypertension	754 (52.4)	720 (51.7)	34 (72.3)	0.020
Malignancy	131 (9.1)	123 (8.84)	8 (17.0)	0.055
Abnormal liver function tests†				
ALT	289 (64.8)	272 (65.8)	17 (52.2)	0.188
AST	420 (94.1)	390 (94.0)	30 (95.7)	0.748
AKP	205 (46.0)	189 (45.5)	17 (52.2)	0.537
TP	247 (55.8)	232 (56.4)	15 (47.8)	0.426
Albumin	357 (80.9)	336 (82.2)	21 (65.2)	0.046
TB	57 (12.7)	51 (12.3)	6 (17.4)	0.483
DB	105 (29.0)	98 (29.3)	7 (25.0)	0.681

Liver injury‡	446 (50.5)	415 (49.7)	31 (65.7)	0.065
Time to development of liver injury, mean±SD, days	3.3±4.4	3.3±4.4	3.2±3.9	0.968

Abbreviations: SD, standard deviation; CLD, chronic liver disease; SD, standard mean; ALT, alanine aminotransferase; AST, aspartate aminotransferase; ALP, alkaline phosphatase; TP, total protein; TB, total bilirubin; DB, direct bilirubin.

†: The number and percentage for all the lab values are for patients with abnormal lab values (i.e., above the normal upper limit).

‡: Liver injury was defined as any parameter above the normal upper limit (including ALT, 6-45 U/L; AST, 10- 42 U/L; ALP, 34-104 U/L; TP, 6.0-8.0 G/DL; albumin, 3.5-5.0 G/DL; TB, 0.2-1.3 MG/DL; DB, 0-0.3 MG/DL), based on the worst liver function test result recorded during hospitalization.

Supplementary Table S4. Postmortem liver findings in eight COVID-19 patients

Patients	Age, y	Gender	History of Chronic Liver Disease	History of Smoking, Alcohol consumption	Comorbid conditions	AKP (U/L)	Albumin (g/dL)	AST/ALT (U/L)	Liver Pathology Findings
1	73	Female	Not available	Active smoker, Alcohol consumption	Not known	Not available	Not available	Not available	No specific pathologic findings
2	63	Female	No	Former smoker	Advanced stage of ALL/Lymphoma, chemo	84	3.2	14/15	Portal triads with minimal lymphocytic infiltrate , focal infiltrate
3	61	Female	No	no	Asthma, CVA, carotid artery dissection, hyperlipidemia hypothyroidism	193	2.3	54 /26	Portal triads with minimal lymphocytic infiltrate, rare fibrin thrombi in portal veins
4	74	Male	No	Former smoker	diabetes, hypertension, CAD, MRSA bacteremia, PV endocarditis	73	2.3	14/08	Portal triads with minimal lymphocytic infiltrate
5	77	Male	No	no	DVT/PE, hypertension, gout, cardiac amyloid	169	2.2	64 /38	Portal triads with mild lymphocytic infiltrate, central vein outflow obstruction
6	80	Female	No	Alcohol consumption, active smoking	COPD, CAD, Sjogren's syndrome	35	2.5	29/18	Portal triads with mild lymphocytic infiltrate, central vein outflow obstruction
7	85	Male	No	No	Cerebrovascular accident with residual aphasia, obstructive sleep apnea, aortic regurgitation, acute endocarditis, hypertension, CKD	34	2.5	83/53	Mild portal tiads with mild lymphocytic infiltrate, central vein outflow obstruction with central lobular necrosis
8	50	Male	No	Former smoker; Alcohol consumption	Myeloproliferative neoplasm (JAK2 mutation) complicated by DVT/PE, large gastric and esophageal varices s/p splenectomy and splenorenal shunt	196	2.6	25 /20	Portal triads with mild lymphocytic infiltrate , rare fibrin thrombi
